# Pediatric inhalation injury

**DOI:** 10.1186/s41038-017-0097-5

**Published:** 2017-11-01

**Authors:** Soman Sen

**Affiliations:** Division of Burn Surgery, Department of Surgery, University of California Davis, Shriners Hospital for Children Northern California, Sacramento, USA

**Keywords:** Inhalation injury, Children, Ventilator management, Smoke inhalation, Carbon monoxide

## Abstract

Smoke inhalation injury can cause severe physiologic perturbations. In pediatric patients, these perturbations cause profound changes in cardiac and pulmonary physiology. In this review, we examine the pathology, early management options, ventilator strategy, and long-term outcomes in pediatric patients who have suffered a smoke inhalation injury.

## Background

Smoke inhalation injury causes a significant and often morbid damage to the lungs that can lead to prolonged mechanical ventilation, increased mortality with concomitant injuries, and long-term pulmonary complications. For pediatric patients with a severe burn injury, inhalation injury significantly increases mortality and affects approximately 20 to 30% of patients [1]. A 10-year multicenter review of 850 pediatric burn patients with inhalation injury showed that overall mortality was 16% and that the majority of the patients died from pulmonary dysfunction and sepsis. However, for pediatric burn patients with an inhalation injury who need more than 1 week of mechanical ventilation, mortality increases to 25 to 50% [2]. Additionally, children under age 4 who suffer both a significant burn injury and inhalation injury have a higher risk of death compared to children over age 4 [3]. This may be due to differences in anatomy and physiology in younger children. Younger children tend to have higher resuscitative fluid requirements due to their high body surface area to weight ratio [4]. Thus, hypovolemia due to inadequate resuscitation in the setting of severe inflammation from an inhalation injury and a severe burn injury can lead to death [5]. Additionally, smaller airways can complicate or delay the appropriate securing of an airway and also lead to increased risk of airway obstruction [6]. Also, younger children may not have fully developed immune systems, which may increase the risk of infections and sepsis leading to death [7].

## Review

### Pathology

For enclosed fires, carbon monoxide (CO) and cyanide toxicity is an early and immediate cause of morbidity and mortality due to hypoxia. Smoke generated from burning wood has significantly higher concentrations of CO and aldehydes compared to smoke from accelerants [8]. If synthetic materials are also burned in the fire, hydrogen cyanide (HCN) is released and becomes a significant component of the inhaled smoke [9]. CO has over 200 times the affinity of oxygen for hemoglobin. This causes a decreased perfusion of oxygenated blood to organs and cells leading to organ and cellular damage [10]. Prolonged smoke exposure causes elevated blood levels of CO, which become increasingly toxic, leading to profound hypoxia brain damage and brain death [11, 12]. Cyanide also produces hypoxia at the cellular levels; however, the mechanism differs from carbon monoxide. Cyanide disrupts mitochondrial generation of adenosine triphosphate (ATP) through binding of ferric ions in cytochrome c oxidase. This interrupts the electron transport chain and blocks aerobic cellular metabolism [13].

Initially, in an enclosed fire, heated air is inhaled into the upper airway. However, because of the reflexive glottis closure, the heated dry air cools significantly and causes minimal to no direct damage of the lower airways. In situations where high-humidity hot air is inhaled, such as super-heated steam, prolonged exposure to the air can cause significant direct thermal damage to the upper and lower airways [14]. Upper airway damage occurs from direct thermal injury. Epithelial damage from heated air causes damage similar to thermal skin injury. Erythema and ulcerations develop in the oropharynx above the vocal cord, and significant edema can ensue over the first 24 h after injury [15]. Initially after an inhalation injury, upper airway damage may not manifest clinically, but over the first several hours after injury, hoarseness, stridor, and dyspnea can occur. The progressive edema can be exacerbated by the acute resuscitation for severe burn injury and can comprise the security of the airway, necessitating early establishment of a secure airway [16, 17].

Tracheobronchial damage below the vocal cords occurs from chemical components of inhaled smoke. Bronchoconstriction is triggered in the tracheobronchial tree through activation of neuropeptides from smoke exposure [18]. This in turn causes mobilization and activation of neutrophils resulting in the release of reactive oxygen species and subsequent cellular damage [19]. This damage manifests itself clinically with erythema and inflammation of the mucosal lining of the bronchial tree. The mucosal damage causes an exudative response resulting in copious exudate filling the bronchi [16]. Further mucosal damage occurs from aldehydes, ammonia, aromatic hydrocarbons, sulfur dioxide, and acrolein causing ciliary damage thus inhibiting mobilization of bronchial exudate [16, 20].

Damage to the lung parenchyma is usually a delayed process and usually manifests itself 24 h after the initial injury. Activation of immune systems occurs from inhaled smoke in the tracheobronchial system [21]. The production of reactive oxygen species and subsequent damage triggers further inflammation resulting damage, obstruction, and collapse of alveoli [22]. This causes edema and consolidation of the pulmonary parenchyma and clinically manifests as ventilation and perfusion mismatches [23]. With the extensive damage to mucociliary function, copious exudate, consolidation, and collapse of alveoli, smoke particulate clearance is impaired. This causes further and persistent inflammation and parenchymal damage [24, 25].

### Diagnosis

Diagnosis of inhalation injury starts with obtaining information about the physical circumstances of the incident. Patients found in enclosed fires, such as a building or a house, are at high risk of having inhaled some smoke. Additionally, fires that occur in manufacturing or storage facilities may have produced smoke that is high in content of toxic-inhaled compounds such as CO, cyanide, aldehydes, and acreolin [26]. For pediatric patients, agitation and confusion may be caused by smoke inhalation, injury, or fear. Physical signs such as singed nasal hairs, smoke soot on the nose and face, and soot in the oropharynx are non-specific signs of smoke exposure [27]. Patients may also exhibit signs of respiratory distress such as stridor, dyspnea, hoarseness, and wheezing [6].

Chest x-rays often do not provide useful information immediately due to the delayed pathologic changes that occur with smoke inhalation. Often changes occur physiologically prior to any changes or evidence of inhalation injury on chest x-rays [28]. Other non-invasive modalities such as xenon lung scanning and computer tomography (CT) can be used to diagnose inhalation injury. Xenon lung scanning with 133xenon isotope can diagnose inhalation injury; however, studies indicate that over 10% of the xenon scans can produce erroneous results [29]. CT scans performed early after an injury show a fine ground-glass appearance to the lungs [30]. However, currently, the most used and reliable method to diagnose the extent and severity of inhalation injuries is fiberoptic bronchoscopy. A recent study comparing inhalation injury diagnostic methods determined that fiberoptic bronchoscopy was the most effective method. Additionally, the severity of injury found with bronchoscopy correlated the best with clinical findings and outcomes [31]. Findings on bronchoscopy can range from mild edema and hyperemia indicating mild injury, severe edema, hyperemia, and soot indicating moderate injury, and ulcerations and necrosis indicating severe injury [32].

### Early management

The early management of patients with inhalation injury centers around assessing and establishing an adequate and stable airway and assessing and treating CO and cyanide toxicity. For pediatric airway management, considerations must be given to age-related anatomic differences and cross-sectional area differences. For younger patients such as infants and toddlers, airway obstruction can occur rapidly due to a number of factors. First, the tracheal anatomy of younger patients is different than adults. The younger patient’s tracheas are more funnel shaped and narrower below the thyroid cartilage compared to adult tracheas [33, 34]. Second, because the cross-sectional area is smaller, any small reduction in the diameter of the trachea exponentially increases the resistance to air passage. Third, younger patients have shorter mandibles, prominent adenoids, and larger tongues, all of which limit the upper airway space [35]. Thus, following an inhalation and severe burn injury, onset of edema coupled with administration of sedatives and pain medication can quickly lead to upper airway collapse [36].

Smoke inhalation injury also exposes patients to several inhaled toxins. Because many pediatric patients are unable to escape from the scene of an enclosed fire, their exposure to these toxins can become significant. Approximately 5% of all acute pediatric inhalation injuries involve inhaling CO [37]. Cyanide toxicity is also a potential contributor to morbidity and mortality in pediatric inhalation injuries. Although cyanide toxicity is rarely reported in children, fatal levels of cyanide are found in over a third of victims of enclosed fires [38].

For CO toxicity, initial management is centered on immediate administration of 100% fractional inspiration of oxygen (FiO_2_) and diagnosis and monitoring of CO toxicity. CO has a 200 times greater affinity for hemoglobin compared to oxygen. With increase exposure to smoke and inhalation of CO, hemoglobin preferentially binds CO forming carboxyhemoglobin (COHb) compounds resulting in hypoxia. Often, the dissolved concentration of oxygen is normal in these settings; however, because of CO affinity for hemoglobin, oxygen is unable to bind to hemoglobin. As a consequence, tissue and cellular delivery of hemoglobin becomes impaired resulting in hypoxia. Additionally, plasma oxygen saturation monitor values may be normal because the infrared wavelength changes for hemoglobin saturated with oxygen versus hemoglobin saturated with CO are the same. COHb can be measured in the blood with arterial blood gas analysis specialized for coximetry. Other methods that can be used to determine the levels of CO toxicity are the CO-oximeters and transcutaneous oxygen measurements [39].

Symptoms of CO toxicity begin to clinically manifest as headaches and confusion at COHb levels of 15 to 20%. At COHb levels of 20 to 40%, patients are often disoriented and may complain of visual disturbances. At COHb levels of 40–60%, patients may become combative or obtunded. COHb level above 60% lead to death in the majority of patients [16]. If suspicions are high for CO exposure, then prompt administration of 100% FiO_2_ will immediately lower the levels of COHb. The half-life of COHb is 60 min when 100% FiO_2_ is administered compared to 5 h on room air oxygen concentrations. Thus, if a patient has a COHb level of 20%, administration of 100% FiO_2_ will reduce COHb to 10% in 60 min [40]. Patients should remain on 100% FiO_2_ until COHb levels return to normal [41]. Hyperbaric oxygen (HBO) also has utility in treating CO poisoning in children [42]. HBO administered at 2.5 atm reduces the half-life of COHb to 20 min. However, HBO has some clinical limitations. Patients are placed in sealed tanks that can only accommodate one other person. This limits acute treatment for other injuries such as burn injuries that are often present in patients with inhalation injuries [43]. Some small studies have demonstrated some efficacy for HBO compared to normobaric oxygen [43]. However, many of these studies have significant design flaws and conclusive evidence does not exist that supports the use of HBO for CO poisoning [44].

HCN is the gaseous form of cyanide and can be a significant component of inhaled smoke from structural fires. Clinically, small amounts of cyanide are metabolized in the liver. However, more abundant and faster absorption of hydrogen through the lungs in inhalation injury overwhelms hepatic metabolism of cyanide leading to toxic levels [45]. Clinical manifestations of cyanide toxicity include neurologic deficits, persistent and unexplained acidosis, and serum lactate greater than 8 mmol/L [46]. Many clinical symptoms are hard to isolate to cyanide toxicity due to concomitant burn injuries. In particular, confusion and agitation in pediatric patients is often present due to age-related anxieties and pain from the burn injury [27]. Other signs of cyanide toxicity are similar in both pediatric and adult patients. These signs include persistent hypotension, cardiac arrthymias, persistent metabolic acidosis, decreased serum or mixed venous oxygen consumption, and persistently increased lactate. These signs are consistent with the profound cellular hypoxia that can occur following cyanide toxicity, and treatment for cyanide toxicity should be considered if these symptoms occur and clinical suspicions are high [47]. There are several methods to diagnose cyanide toxicity. Non-direct testing includes serum lactate levels, anion gap, and methemoglobin concentrations [48]. Cyanide levels can also be directly measured in the blood. Levels of 0.5 to 1 mg/dL cause flushing and tachycardia, levels between 1 and 2.5 m/dL can induce delirium and coma, and levels above 3 mg/dL cause brain death [48]. For treatment, hydroxocobalamin has shown some efficacy in lowering cyanide levels. Hydroxocobalamin is a cobalt compound that binds to cyanide and transforms cyanide to a non-toxic derivative [49]. In the clinical settings, several limited studies have shown efficacy for hydroxocobalamin in the setting of inhaled cyanide toxicity; however, current evidence does not support empiric administration [50]. Sodium thiosulfate can also be used to lower cyanide levels and treat toxicity. Sodium thiosulfate binds to cyanide to donate a sulfur group to form a less toxic compound thiocyanate. However, because of its rapid onset, safety, and efficacy, hydroxocobalamin has been touted as the antidote of choice for cyanide toxicity [49, 51].

### Ventilator management

Ventilator management in pediatric inhalation injury should focus on providing adequate gas exchange while minimizing ventilator-induced injury [52]. Infants and toddlers have a much higher oxygen consumption and carbon dioxide production than adults and thus require a much higher respiratory rate [53]. In parallel, aggressive pulmonary hygiene should be immediately implemented. Due to the exudate reaction triggered by smoke inhalation, airways and functional units of the lungs can become obstructed and filled with exudative debris. Additionally, the impaired mucocilliary function further limits clearance of mucous and exudate [19]. This coupled with pulmonary edema can further exacerbate poor gas exchange. Pediatric patients with concomitant inhalation and burn injury are particularly susceptible to developing pulmonary edema. This may be due to “fluid creep” that may occur during resuscitation [54]. “Fluid creep” is the administration of intravenous fluid during burn resuscitation, that is a higher volume than the Parkland formula calculation. Pediatric patients are particularly susceptible to this phenomenon. This may be due to pre-admission fluid administration, inaccurate measurements of body surface area, inaccurate measurements of weight, inaccurate estimates of the extent of burn injury, or a combination of these factors [55].

Younger patients, infants and toddlers, are at higher risk of airway obstruction due to smaller airways and less develop tracheobronchial tree [56]. Inhaled beta-receptor agonists may help in decreasing bronchospasm and improve airway obstruction following burn injury. In a small pediatric inhalation injury study, nebulized epinephrine was administered on admission and given every 4 h for 7 days. This group was compared to patients who only received standard of care. The investigators found that nebulized epinephrine could be given safely but did not find any significant differences in the number of days of ventilation or functional outcomes [57]. Continuously inhaled albuterol may also be beneficial. An ovine model of inhalation indicated that 20 and 40 mg per hour of continuously inhaled albuterol resulted in decreased peak airway pressure, decreased pause pressure, and increased compliance [58]. However, to date, there are currently no clinical investigations that support the use of continuous albuterol in pediatric inhalation injury.

Mechanical ventilation in pediatric inhalation injury patients can be challenging due to physiologic and pathologic changes that occur. Damage from smoke inhalation results in pulmonary parenchymal damage and causes decreased pulmonary compliance and increased airway resistance [59]. As a consequence, ventilator management strategies for pediatric inhalation injury patients have centered on decreasing further damage from ventilator-induced barotrauma [60]. Many ventilator modes can be used for pediatric inhalation injury patients. A survey of pediatric burn centers found that a variety of ventilator modes are used from conventional pressure and volume mode ventilators to high-frequency oscillatory and jet ventilators [61]. For conventional ventilators, controversy exists as to the optimal tidal volume settings. Since the description of the mortality benefits of low tidal volume ventilation for acute respiratory distress syndrome, many centers have decreased the tidal volume limits for pediatric inhalation injury [62]. However, pediatric patients with a burn injury were excluded from this study and a consensus on appropriate tidal volumes for pediatric patients with acute respiratory distress has not been achieved [63]. A recent retrospective study compared clinical outcomes between high tidal volume (15 mL/kg) and low tidal volume (9 mL/kg) settings in pediatric burn patients with inhalation injuries. The investigators found that high tidal volumes decreased ventilator days and atelectasis. However, the high tidal volume group suffered significantly more pneumothoraxes compared to the low tidal volume group. Additionally, there was no significant difference in mortality. [64]. Thus, while high tidal volumes may improve pulmonary function, current evidence does not support this strategy in pediatric patients with inhalation injury. Prospective comparisons for short- and long-term outcomes between high and low tidal volumes in this patient population are needed to resolve this important issue.

Non-conventional ventilators have been used with some reported success in pediatric inhalation injury. High-frequency percussive ventilation (HFPV) provides high-frequency small tidal volumes in combination with low-frequency breathing rates [65]. This is combined with a low-pressure circuit to maintain airway patency and limit volumetric trauma. In burn patients, this mode of ventilation may improve gas exchange and airway pressures compared to the conventional ventilator modes [66]. A small study comparing pediatric inhalation injury patients on HFPV versus conventional ventilation indicated that patients in the HFPV group had fewer pneumonias, lower peak inspiratory pressure, and decreased work of breathing [67]. High-frequency oscillatory ventilation (HFOV) also has shown some success in treating pediatric burn patients. High-frequency oscillatory ventilation provides high respiratory rates with very low tidal volumes. This creates a high flow of oxygen without a marked increase in airway pressures [68]. The efficacy of HFOV was studied in a small sample of pediatric burn patients of which half were injured in a house fire. The study indicated that HFOV might significantly improve oxygenation. Thus, while there are a number of effective mechanical ventilation modes, larger prospective studies are needed to determine if any of these modes are superior clinically.

Extracorporeal membranous oxygenation (ECMO) may be used in cases of severe pulmonary failure. A review of the Extracorporeal Life Support Organization registry found 36 pediatric burn patients treated with ECMO from 1999 to 2008. Seventeen patients underwent venovenous ECMO, and 19 underwent venoarterial ECMO. Overall survival was 53%. Eleven patients were placed on HFOV, and 18 were placed on conventional ventilation. Of those placed on conventional ventilation, 8 survived. For the patients placed on high-frequency oscillatory ventilation, 7 survived. There were 7 patients who did not have information regarding the type of mechanical ventilation [69].

### Long-term outcomes

Inhalation injury in pediatric populations may not affect self-reported disability or quality of life. One hundred and thirty-five pediatric burn patients were assessed for disability using the World Health Organization Disability Assessment Scale II. The investigators found that there were no differences in long-term disability between patients who suffered an inhalation injury and a burn injury compared to those who suffered only a burn injury. Quality of life in these same patients was assessed using the Burn Specific Health Scale-Brief. As with disability, the investigators found no difference in long-term quality of life between patients with inhalation and a burn injury and patients with a burn injury alone [70].

## Conclusions

Pediatric inhalation injury has high morbidity and mortality when combined with a burn injury. Considerations must be made for age-related differences in exposure, anatomy, and physiology in order to provide optimal and efficient treatment. Early diagnosis and initiation of treatment can mitigate serious and dire consequences. Prospect studies are needed in a number of diagnostic and treatment areas to determine benchmark treatment strategies.

## Abbreviations

ATP: Adenosine triphosphate
CO: Carbon monoxide
COHb: Carboxyhemoglobin
CT: Computer tomography
ECMO: Extracorporeal membranous oxygenation
HBO: Hyperbaric oxygen
HCN: Hydrogen cyanide
HFPV: High-frequency percussive ventilation
HFOV: High-frequency oscillatory ventilation
